# Hepatitis E and Pregnancy: An Unholy Alliance Unmasked from Kashmir, India

**DOI:** 10.3390/v13071329

**Published:** 2021-07-09

**Authors:** Mohammad Sultan Khuroo

**Affiliations:** Digestive Diseases Centre, Dr. Khuroo’s Medical Clinic, Srinagar, Jammu and Kashmir 190010, India; khuroo@yahoo.com; Tel.: +91-9906591044

**Keywords:** hepatitis E, hepatitis E virus, genotypes, pregnancy, epidemic hepatitis, sporadic hepatitis, acute liver failure, fetus, neonate, delivery, hepatitis E vaccine

## Abstract

The adverse relationship between viral hepatitis and pregnancy in developing countries had been interpreted as a reflection of retrospectively biased hospital-based data collection by the West. However, the discovery of hepatitis E virus (HEV) as the etiological agent of an epidemic of non-A, non-B hepatitis in Kashmir, and the documenting of the increased incidence and severity of hepatitis E in pregnancy via a house-to-house survey, unmasked this unholy alliance. In the *Hepeviridae* family, HEV-genotype (gt)1 from genus *Orthohepevirus* A has a unique open reading frame (ORF)4-encoded protein which enhances viral polymerase activity and viral replication. The epidemics caused by HEV-gt1, but not any other *Orthohepevirus* A genotype, show an adverse relationship with pregnancy in humans. The pathogenesis of the association is complex and at present not well understood. Possibly multiple factors play a role in causing severe liver disease in the pregnant women including infection and damage to the maternal-fetal interface by HEV-gt1; vertical transmission of HEV to fetus causing severe fetal/neonatal hepatitis; and combined viral and hormone related immune dysfunction of diverse nature in the pregnant women, promoting viral replication. Management is multidisciplinary and needs a close watch for the development and management of acute liver failure. (ALF). Preliminary data suggest beneficial maternal outcomes by early termination of pregnancy in patients with lower grades of encephalopathy.

## 1. Historical Background

Space-time clustering of events in which people fall acutely ill with jaundice, quickly slip into a coma and die, is an alarming situation, more so when the victims are mostly or exclusively pregnant women [1].

The association between epidemics of jaundice and pregnancy has long been reported in scientific literature. The earliest recorded epidemic of jaundice with high mortality in pregnant women was reported from the French Caribbean colony Martinique in the year 1858 [2]. A strange disease had struck the island which left 24 women dead, and 20 of these were pregnant. All the deceased pregnant women had delivered stillborn babies. None of the jaundiced soldiers, all males, in the nearby garrison had died. Another notable epidemic of jaundice occurred in Paris in 1871. Deaths occurred exclusively in gestating women and autopsies revealed acute yellow atrophy of the liver as the cause of death [3]. Over the ensuing decades until 1946, many countries in Europe recorded several epidemics of jaundice with high death rates in pregnant women [1]. These epidemics also reported high rates of fetal and neonatal deaths as a result of abortions, premature deliveries, miscarriages, and stillbirths in both dead and surviving pregnant women. All these outbreaks were likely to have been related to hepatitis E; however, no serological tests of sera from such outbreaks are available. With improvement in economic conditions and clean water supplies, epidemic disease with high mortality in pregnant women is no longer reported from industrialized countries [4].

## 2. Controversy Over Data—West versus East

In the latter part of the last century, viral hepatitis and pregnancy was a matter of investigation and controversy [5]. The data published from various industrialized countries of Europe, North America and Australia had indicated that pregnancy does not increase the severity of disease and /or susceptibility to infection [6,7,8,9,10]. This was expected as hepatitis E was not endemic in such regions of the world [11]. However, several reports from developing countries in Asia especially India, Iran, and the Middle East, had shown that there is increased severity and mortality from viral hepatitis among pregnant women [12,13,14,15,16]. These reports were based on retrospective analysis of hospital admissions and were interpreted in some studies as a reflection of biased hospital-based data collection, due to the fact that these data predated the discovery of HEV [17].

## 3. Unmasking the Unholy Alliance

**Figure 1:**
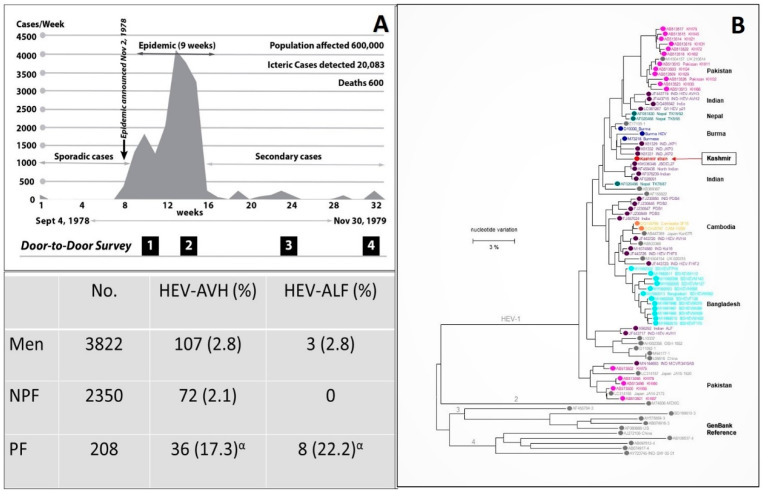
Gulmarg Kashmir Epidemic, 1978–1979. (**A**) Upper panel. The epidemic curve with the weekly occurrence of hepatitis E cases. The region had an open-source water supply from a canal (Ningli Nallah) which originates from the Alpather Lake situated at the foot of Apharwat Peaks, Gulmarg. After passing through mountains as the world-famous *Sharanz waterfall,* the stream crosses the valley to join the Wular lake. The canal along its route is used for multiple purposes including drinking water, linen washing, swimming, fishing, and sewage and garbage disposal, and thus stays highly polluted. Lower panel. The data on incidence and severity of viral hepatitis in men (15–45 years), NPF (nonpregnant females; 15–45 years) and PF (pregnant females) were collected by four door-to-door surveys done at 4 to 6 week intervals during the epidemic. (**B**) Kashmir strain of HEV (Pinglina epidemic, 1993–94, Kashmir, India [20].). Unrooted phylogenetic tree generated by Maximum Likelihood method using MEGA software (version 10.1.8), on the basis of 326 bp sequences of HEV ORF1 genomic. Reference sequences of different HEV genotypes from GenBank are shown in gray filled circles, while country specific HEV sequences are shown in color filled circles. Kashmir strain of HEV was of genotype 1 with 94.6% homology with the Burmese isolates of HEV (Courtesy Saleem Kamili & Xia, Guo-Liang, both at CDC, Atlanta, Georgia). α = 6 pregnant women died.

The association between viral hepatitis and pregnancy was delineated with the discovery of HEV from an epidemic of viral hepatitis in Kashmir, India [18] and the reporting of the true incidence and severity of the disease in pregnancy based via a prospective door-to-door study [19] (Figure 1).

### 3.1. Discovery of Hepatitis E

The discovery of HEV started with studies carried out on a large-scale epidemic of viral hepatitis in Gulmarg-Kashmir, India in November 1978 [18]. Investigating an epidemic of the size had posed problems of harsh weather, primitive healthcare facilities, a highly compressed time period, lack of funding, and hesitancy of medical manpower to join the team due to fear of personal health risks. To face the challenges, a team of 500 healthcare workers (local inhabitants) opted to reside in the epidemic region, support the primitive healthcare facilities, offer care to the needy at the doorstep, and record every case of hepatitis from the community (Figure 1). Over 9 weeks, 20,083 cases of acute viral hepatitis (AVH) with 600 fatalities were recorded. The epidemic curve was highly compressed with the occurrence of up to 4000 icteric cases per week. The disease selectively affected young adults and presented as acute hepatitis syndrome with cholestatic features in around 20% of the affected individuals. Liver histology showed portal and lobular hepatitis with necrosis and ballooning degeneration and Kupffer cell hyperplasia, with a subset of patients showing distinctive features in the form of intracanalicular bile stasis and rosette formation of hepatocytes as a dominant feature. All patients who survived recovered and none developed chronic liver disease [21]. IgG anti-HAV, as a marker of past exposure and immunity to hepatitis A virus (HAV), was reactive in all patients, while none was seropositive for IgM anti-HAV, HBsAg, and IgM anti-HBc. It was postulated that this epidemic of enterically transmitted non-A, non-B hepatitis (ET-NANBH), was caused by an agent different from post-transfusion non-A, non-B hepatitis (PT-NANBH), later identified as hepatitis C virus (HCV) [22].

Mikhail Balayan self-infected himself via stool samples from an outbreak of hepatitis that occurred among Soviet troops in Afghanistan. He and his team identified virus-like-particles (VLPs) in his stool samples [23]. Reyes et al. isolated a cDNA from the virus responsible for ET-NANBH in a stool from Burma [24]. Tam et al. cloned and sequenced the full length of HEV [25] and Yarbough et al. developed a serological test for diagnosis of HEV infection [26].

After these developments, sera samples (n = 114) collected during the Gulmarg Kashmir epidemic 1978–79, 71% tested positive for IgG anti-HEV and 75% of these were reactive for IgM anti-HEV, confirming HEV as the causative agent of the epidemic [27]. From 1978 to 2013, ten epidemics of viral hepatitis were reported from Kashmir, India [18,19,28,29,30]. Sera from all ten epidemics were tested for IgG anti-HEV, IgM anti-HEV and HEV RNA and the epidemics were confirmed to be caused by HEV [20,30,31,32]. Partial genomic sequencing of HEV from two such epidemics, namely the Jammu epidemic 1988 and the Pinglina epidemic of 1993–94, were carried out. Both virus strains belonged to HEV-gt1 and had a homology of 94.6%–96.8% to the Burmese strain of HEV (Figure 1) [20,33]

### 3.2. Incidence and Severity of Hepatitis E in Pregnancy

During the Gulmarg-Kashmir epidemic of 1978–79, a prospective study was done to define the incidence and severity of hepatitis E in pregnant women compared with non-pregnant women of child-bearing age and men (15–45 years) [19]. The data were collected in Block Sopore, consisting of 15 villages with a population of 16,620. The four door-to-door surveys were conducted at 4-to-6-week intervals to identify every new case of hepatitis (Figure 1). A total of 275 cases of hepatitis E were recorded. Thirty-six (17.3%) of the 208 pregnant women were infected with HEV as compared to 71 (2.1%) of 3350 nonpregnant women and 107 (2.8%) of 3350 men. The incidence of disease in the first trimester (3/34; 8.8%), second trimester (15/77; 19.2%), and third trimester (18/97; 18.6%) was higher when compared to that in nonpregnant women and men. Acute liver failure (ALF) developed in 22.2% (8/36) of pregnant women with HEV infection, as compared to 2.8% (3/107) of men and no (0/71) nonpregnant women. Nine deaths had occurred, six in pregnant women and three in men. The case fatality rate of HEV infection was 16.6% (6/36) in pregnant women and 2.8% (3/107) in men. None of the nonpregnant women with HEV infection died. None of the 18 pregnant women with HEV infection in their first and second trimester developed ALF, while 8 (44.4%) of 18 pregnant women with HEV infection in the third trimester developed acute hepatic failure with six deaths. The case fatality rate of HEV infection in the third trimester of pregnancy was 33.3% (6/18). These data were conclusive that, during epidemics, HEV infection showed increased incidence and severity in pregnancy. The incidence was higher in all three trimesters as compared to HEV- infected men and non-pregnant women, while the increased severity of disease was restricted to the third trimester of pregnancy.

## 4. Hepatitis E

**Figure 2:**
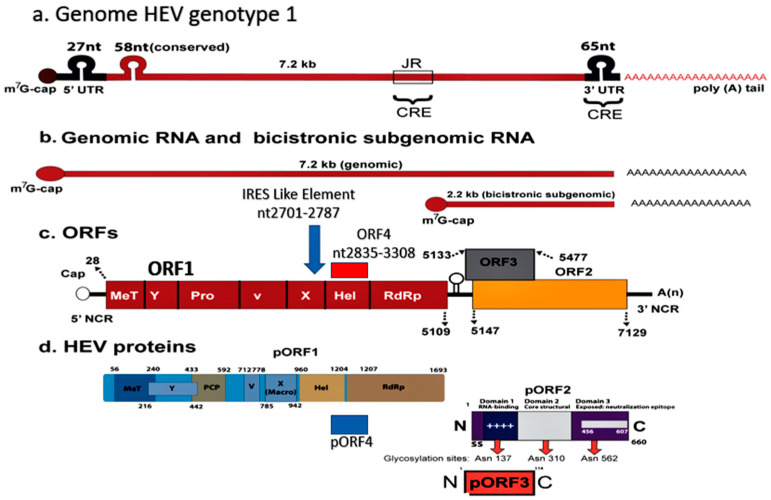
Hepatitis E virus Genotype 1. Genomic organization. (**a**) The hepatitis E virus genome; (**b**) Genomic RNA and bicistronic sub-genomic RNA; (**c**) Four Open reading frames (ORFs) and (**d**) Four encoded proteins (pORF1, pORF2, pORF3, and pORF4). ORF4, spanning nt2835-3308 and overlapping ORF1 in present in HEV genotype 1 alone, and its protein expression is regulated via an IRES-like RNA element (nt2701-2787). ORF4 encodes a protein (pORF4) of 124 aa, which functions to enhance viral polymerase activity and promote viral replication and is indispensable for the HEV genotype 1 life cycle.

Hepatitis E is one of the five main forms of viral hepatitis and is caused by infection with HEV [34]. HEV is a group of viruses in the family *Hepeviridae* [35]. These viruses are quasi-enveloped, have an icosahedral shape with 20 faces, spherical geometry with surface spikes and indentations, and T = 1 symmetry. Genomes are linear, non-segmented, 7.2 kb in length, and have three open reading frames (ORFs), namely ORF1, ORF2 and ORF3 [36]. HEV has marked genetic heterogeneity and is divided into two genera, namely *Orthohepevirus* and *Piscihepevirus* [37]. *Orthohepevirus* has four species A, B, C and D. *Orthohepevirus A* has eight genotypes (gt). HEV-gt1 and HEV-gt2 infect humans alone. An additional ORF4, spanning nt2835-3308 and overlapping with ORF1, is present in HEV-gt1 alone and its protein expression is regulated via an IRES-like RNA element (nt2701-2787). ORF4 appears to cause endoplasmic reticulum (ER) stress which may be involved in promoting viral replication [38,39] (Figure 2). As C-terminal 19 amino acids are absent in around half of the genomes, only the N-terminal 124 amino acids of pORF4 can interact with other viral and host proteins. This protein functions to enhance viral polymerase activity and promote viral replication and is indispensable for the HEV-gt1 life cycle [40]. HEV-gt3 and HEV-gt4 are highly divergent and have been isolated from several animals including pig, wild boar, deer, mongoose, rabbit, goat, horse, bottlenose dolphin, and sheep (HEV-gt3); and pig, wild boar, cattle, cow, sheep, goat, and yak (HEV-gt4) [41,42]. HEV-gt5 and HEV-gt6 infect wild boar in Japan and HEV-gt7 and HEV-gt8 infect dromedary and Bactrian camels, respectively [43]. HEV-gt3 and HEV-gt4 from pigs and possibly rabbits are zoonotic, and isolated cases of HEV-gt7 infection in humans have been reported [36,37]. *Orthohepevirus B* has four genotypes, infects birds, primarily chicken, and causes hepatitis-splenomegaly syndrome and big liver-spleen disease in chicken. *Orthohepevirus C* causes infection in rats, voles, mice, and ferret and *Orthohepevirus D* infects bats. Cases of rat HEV infection in humans have been reported [44,45]. Genus *Piscihepevirus* includes a single species containing cutthroat trout virus [37]. Isolates from moose, fox, and little egret have remained unassigned as yet [35,37]. A recent study showed that people in households with seropositive goats were more likely to be seropositive themselves than persons living in households with seronegative goats [46]. Hepatitis E is a global disease and was estimated to have caused 20.1 million incident infections in the year 2005, out of which approximately 3.4 million infections were symptomatic with around 70,000 deaths and 3000 stillbirths [47]. Hepatitis E has distinctive epidemiological and clinical characteristics in developing countries, which contrast sharply with those in the industrialized world [48,49]. Based on disease pattern and prevalence and genotype distribution, four hepatitis E epidemiological patterns are seen [11]. The first is an hyperendemic zone that encompasses many countries in the Indian subcontinent, Southeast Asia, Central Asia, many regions in Africa, and Mexico. Here HEV-gt1 and HEV-gt2 present as an epidemic and an endemic disease. The second is an endemic zone that involves several countries in the Middle East and South America and some regions of Southeast Asia (Singapore). HEV-gt1 causes one-fourth of sporadic hepatitis and ALF in these countries. Epidemics of hepatitis E do not occur. The third epidemiological zone is limited to Egypt wherein HEV-gt1 infections occur at young age similar to hepatitis A. Recent studies have shown that symptomatic HEV infections do occur in elderly people and those with pre-existing liver diseases. [50,51,52]. Seroprevalence studies have shown high occurrence of asymptomatic infections in the general population as well as pregnant women [53,54]. This could be related to zoonotic food-borne exposure to HEV-gt3 through cow’s milk, goat milk and goat liver [46,55,56]. It was also observed that naturally acquired immunity appear to protect HEV-exposed subjects from contracting HEV infection during an epidemic [57]. HEV-gt3 and HEV-gt4 cause sporadic autochthonous zoonotic food-borne infection in industrialized countries, which constitute the fourth zone. HEV-gt3 has been reported from many European countries, North and South America, Russia, Japan, and Australia; while HEV-gt4 is prevalent in many regions of China, several Southeast Asian countries, Japan, and a few countries in Europe.

## 5. Epidemic Hepatitis E and Pregnancy

Several large-scale water-borne epidemics of hepatitis have been reported from many regions of the developing world [1] (Table 1). Initially, these epidemics were designated empirically as ET-NANBH based on serological testing for HAV and HBV infections. Later, once serological testing for HEV infection was available, most such epidemics were found to be caused by HEV. HEV strains, once characterized, were all of HEV-gt1 [58,59]. Epidemic HEV infections had several features in common including occurrence in young adults (15–45 years), significant cholestatic features, self-limiting disease, and high mortality in pregnancy.

**Table 1:** Epidemics of hepatitis E with number of recorded cases, case fatality rate (overall and in pregnant women) and relationship with hepatitis E virus genotypes.

Region Year [References µ]	Number of HEV Infections	CFR (%)		HEV Genotypes
		Overall	Pregnancy	
Kashmir 1978–2013 [18,19,20,28,29,30,32,33]	55,563	3.19	22.0	HEV-gt-1
New Delhi 1956 [58,60,61]	29,300	0.9	10.5	HEV
Kanpur 1991 [62]	79,091	0.06	27.0	HEV
Azamgarh 1982 [63]	152	12	39.0	ENANBH
Kolhapur 1981 [58,64,65]	1169	0.25	8.33	HEV-gt1a
Islamabad 1997 [66,67,68]	3827	0.2	11.4	HEV-gt1b
Rangoon 1985 [25,69]	399	3.5	12.0	HEV-gt1
Kathmandu 1981 [70,71]	12,000	-	21.0	HEV-gt1
Kathmandu 1987 [71,72]	7405	0.41	24.65	HEV-gt1
Bangladesh 2008 [73,74]	4198	0.47	19.0	HEV-gt1
Bangladesh 2010 [74,75]	2162	0.55	25	HEV-gt1
Turkmenistan 1985 [76,77]	16,175	0.12	27.4	HEV-gt1
Uzbekistan 1985 [78,79]	12,000	-	7.1	HEV-gt1
Xinjiang 1986 [80,81]	120,000	0.59	13.3	HEV-gt1
Indonesia 1991 [82]	1688	1.78	26.3	HEV
Algeria 1980 [83,84]	788	1.39	100	HEV-gt1
Sudan 2006 [85,86]	253	13.5	31.1	HEV-gt1
Djibouti 1998 [87,88]	42	9.5	33.3	HEV-gt1
Central African Republic 2002 [87,89]	715	0.55	14.28	HEV-gt1
Somalia1993 [87,90]	11,413	2.9	13.8	HEV-gt1
Kenya 1991 [91,92]	1702	3.70	14.28	HEV-gt1
Sudan 2004 [85,87]	2621	1.71	31.14	HEV-gt1
Uganda 2007 [93]	4789	1.50	6.87	HEV-gt1
Mexico 1986 [94]	223	1.35	0	HEV-gt2
Namibia 1995 [95]	>600	0.50	1 death β	HEV-gt2
Namibia 1983 [96,97]	201	3.48	85.7	HEV-gt1
Nigeria 2018 [98,99]	146	1.37	8	HEV-gt1 & HEV-gt2
Central African Republic 2008 [100]	222	1.8	20	HEV-gt1
Chad 2004 [101]	989	3.0	-	HEV-gt1 & HEV-gt2
Namibia 2017 [102]	7247	0.80	6.00	HEV
Chad 2016 [103]	1293	0.69	3.16	HEV-gt1

CFR = Case fatality rate, gt = genotype, ENANBH = Epidemic non-A, non-B hepatitis, ^β^ = number of pregnant women not mentioned in calculating CFR. µ = The references include reports of the epidemic and further studies on the stored samples to characterize the epidemic and the HEV genotypes.

From 1978 to 2013, 10 large-scale water-borne epidemics of hepatitis E were recorded in Kashmir [18,19,20,28,29,30,32,33]. A total of 55,563 persons had contracted the disease and 1775 died with a case fatality rate (CFR) of 3.19%. CFR of HEV in pregnant women during these epidemics was 22%. A retrospective analysis of sera from a large-scale water-borne epidemic that occurred in Delhi in 1955–56 revealed that the epidemic was caused by HEV [58,60,61]. This epidemic affected an estimated 29,300 patients with 266 deaths. Overall, CFR was 0.9% and CFR in pregnancy was 8.5%. A massive epidemic of hepatitis E involving an estimated 79,000 cases visited Kanpur, UP, India in 1992, with a CFR of 27% in pregnant women [62]. Several other outbreaks have been reported from other parts of India [63,64,104,105,106], Pakistan [66,67,68,107], Burma [25,69], Nepal [70,71,72], and Bangladesh [73,74,75,108,109], and all of these showed high CFR in pregnant women [58]. Several regions in Central Asia namely Turkmenistan [76,77], Uzbekistan [78,79], Tajikistan [110], and Kirgizstan [111], have been hit by epidemics of viral hepatitis caused by HEV-gt1. These epidemics affected between 10,000 and 30,000 persons and saw high mortality in pregnant women with CFR ranging from 7% to 27%. Xinjiang region in the northwest of China recorded a massive outbreak of viral hepatitis affecting 120,000 people (mostly Uighurs) in the autumn of 1987–88. CFR in pregnant women was 13% [80]. The outbreak was later confirmed to be caused by HEV-gt1 [81]. Regions of South-East Asia, namely Indonesia [82] and Vietnam, [112] have reported several epidemics of hepatitis E with a high fatality rate of up to 26% in pregnant women. Several outbreaks caused by HEV-gt1 have been reported from many regions of Africa including Somalia [87,90], Algeria [83,84,113], Côte d’Ivoire [114], Botswana [115], Djibouti [87,88] and Central African Republic [87,89], with higher fatality in pregnant women. Of late, outbreaks of hepatitis E in refugee camps among displaced people in several African countries including Somalia [87,90,116], Kenya [91,92], Sudan [85,86] and Uganda [93,117] have occurred. All these epidemics have reported higher death rates in pregnant women.

HEV-gt2 was the incriminating agent for two outbreaks of hepatitis that occurred in two villages 70 km south of Mexico City in 1986 [94]. Of the 223 cases, three women died with an overall CFR of 1.35%. Higher fatality in pregnant women was not reported. The epidemic caused by HEV-gt2 from Namibia in 1995 also did not report higher deaths in pregnant women [92,95,102]. However, a previously reported epidemic from Namibia in 1983 was caused by HEV-gt1 [96,97] and of the 201 cases six of the seven deaths were seen in pregnant women. Epidemics of hepatitis in Nigeria [98,99], Central African Republic [100], and Chad [101,103] were caused by both HEV-gt1 and HEV-gt2 and all reported higher deaths in pregnant women. Few cases of HEV-gt3 and HEV-gt4 infections reported in pregnant women have not been associated with severe disease or deaths [118,119]. An HEV-gt3 outbreak on a cruise ship causing 33 infections did not cause higher mortality in pregnant women [120]. HEV-gt3 and HEV-gt4 are prevalent in industrialized countries and have not been associated with higher mortality in pregnant women [121]. Thus, higher mortality of epidemic hepatitis E in pregnancy is genotype-specific and associated with HEV-gt1 and not with other genotypes causing human infections, namely HEV-gt2, HEV-gt3, and HEV-gt4. However, adverse pregnancy outcomes including miscarriage and stillbirths have been reported in pregnant rabbits experimentally infected with HEV-gt3 and HEV-gt4 [122,123,124].

## 6. Sporadic Hepatitis E and Pregnancy

In 1983, 293 patients with acute sporadic viral hepatitis, of whom 155 cases were caused by HEV infections were reported [20,125]. The mode of transmission was enteric, mostly based on person-to-person contact. The disease occurred in young adults with relative sparing of children. The clinical profile resembled AVH with cholestasis in a subset of patients. The disease was self-limiting and none of the patients developed chronic hepatitis or cirrhosis on follow-up. All these features resembled the epidemic HEV described from Kashmir [18]. The disease occurred in 19 pregnant women. The overall case fatality rate (CFR) was 12.3% and CFR in pregnant women was 35.6%. After this, the etiology, clinical course, and outcome of AVH in 76 pregnant women and 337 non-pregnant women of childbearing age were studied [126]. The prevalence of HEV in pregnant women was 85.5% (65/76 patients) as against 41.5% (140/337) in nonpregnant women. The prevalence of HEV infection was 76.9% (4/13), 88.9% (12/18), 83.8% (23/37), and 100% (8/8) in first, second, third trimesters, and puerperium, respectively. The CFR of HEV infection in pregnant women was 69.2% (45/65) as against 10.0% (14/140) in nonpregnant women. The CFR was 40% (4/10) in the first trimester as against 74.5% (41/55) in the second trimester and beyond. A north Indian study reported HEV as the cause of acute sporadic hepatitis in 82%, 49%, and 57% of pregnant women, nonpregnant women, and men, respectively [127]. Several other studies of acute sporadic viral hepatitis showed a higher prevalence of HEV infections and higher CFR in pregnant women [128,129,130,131,132] (Table 2).

**Table 2:** Prevalence of HEV infection among pregnant women with acute sporadic viral hepatitis.

Author, Year. [References]	Study Material		HEV-AVH (%)		HEV-ALF (%)		HEV Status
	PF	Others	PF	Others	PF	Others	
Khuroo et al., 1983 [125]	27	266α	19 (70.4)	136 (51.1)	6 (31.6)	13 (9.6)	HEV
Nayak et al., 1989 [127]	169	70β	138 (81.6)	34 (48.6)	21 (28.5)		ETNANBH
Jaiswal et al., 2001 [129]	127	146β	83 (65.4)	129 (88.4)	44 (53.0)	17 (13.2)	HEV
Khuroo et al., 2003 [126]	76	337β	65 (85.5)	140 (41.5)	46 (70.8)	14 (10)	HEV
Beniwal et al., 2003 [130]	97	-	46 (47.4)	-	18 (39.1)	-	HEV
Patra et al., 2007 [128]	220	-	132 (60)	-	73 (55.3)	-	HEV

PF = Pregnant females, α = all age groups, β = nonpregnant women of childbearing age.

Regarding HEV genotypes prevalent in acute sporadic hepatitis in India, Arankalle et al. [65] studied 17 HEV isolates (both epidemic and sporadic) from India and found all to be related to various subtypes of HEV-gt1. In another study, Indian swine were found to be infected by HEV-gt4, while all Indian human isolates studied were HEV-gt1 [133]. Gupta et al. [59] studied sequences of 74 patients with acute sporadic hepatitis E from North India and found all the isolates were related to HEV-gt1a. Thus, HEV circulating in India and causing acute sporadic viral hepatitis with higher mortality in pregnancy is also genotype-specific and associated with HEV-gt1.

## 7. HEV-ALF and Pregnancy

Several large series of ALF and its relationship with pregnancy have been reported from India. In one study, amongst 180 patients with ALF [134] forty-nine of the 111 women were pregnant. Seventy-nine patients were related to HEV and the remaining 101 patients were caused by HAV (4 cases), HBV (25 cases), HCV (13 cases), HDV (two cases), drug (one case), and non-A-E agents (56 cases). HEV was the cause of ALF in 47 of the 49 pregnant women as against 14 of the 34 nonpregnant women of childbearing age. Acharya et al. [135] from the All India Institute of Medical Sciences (AIIMS), New Delhi, reported 423 patients with ALF. Of the 223 women, 53 were pregnant. The etiology included HAV (seven cases), HBV (117 cases), HDV (16 cases), NANBH (264 cases), and drugs (19 cases). Thirty-one of the 50 cases from the NANBH group were caused by HEV. Subsequently, 1015 patients with ALF were reported from the same center. 249 of the 647 women were pregnant. HEV was the etiological cause in 342 patients, while 651 patients had non-HEV etiology [136]. HEV was the cause of ALF in 145 of the 244 pregnant women, 100 of the 329 nonpregnant women, and 97 of the 420 men. In another study, HEV was the cause of ALF in 102 of the 139 pregnant women as against 111 of the 181 nonpregnant women (*p* < 0.03) [137]. Kar et al. studied 100 patients with ALF, 50 of whom were pregnant and another 50 nonpregnant women of childbearing age. ALF was caused by HEV in 28 pregnant women against 7 of the 50 nonpregnant women [138]. Sequencing data of all HEV positive sera detected HEV-gt1. These data point to the fact that a substantial proportion of ALF in India is seen in pregnant women and HEV is the dominant etiology.

HEV-ALF in pregnant women starts with prodrome followed by other features of acute viral hepatitis [29]. However, a rapidly evolving devastating illness develops within a short pre-encephalopathy period (5.8 ± 5.3 days), characterized by encephalopathy, cerebral edema with features of cerebellar coning, coagulopathy, and upper GI bleed [134,135,139]. In addition, the occurrence of “Disseminated intravascular coagulation (DIC)” is a distinctive feature of HEV-ALF during pregnancy [140], resembling a Schwartzmann phenomenon.

The prognosis of HEV-ALF in pregnant women has been studied by several investigators [134,136]. The investigators from AIIMS, New Delhi, questioned the worse prognosis of HEV-ALF during pregnancy [136,141]. The authors compared the mortality rates in 249 pregnant women, 341 non-pregnant women, and 425 men, 15 to 45 years of age. The mortality rates in the three groups were 53.8%, 57.2%, and 57.9%, respectively (*p* = 0.572). A prospective study on 180 pregnant women with ALF revealed 79 with HEV-ALF and 101 with non-HEV-ALF [134]. The prognosis in patients with HEV-ALF was better than those with non-HEV-ALF (CFR 51.9% in HEV-ALF versus 84.2% in non-HEV-ALF). Factors predictive of poor prognosis included non-HEV etiology, prothrombin time > 30 s, grade of coma > 2, and age > 40 years and did not include pregnancy per se or duration of pregnancy. The fact that pregnant women acquired ALF more often did not mean that such patients will have higher mortality [142].

## 8. Proposed Hypothesis on Pathogenesis of Mortality in HEV-Infected Pregnant Women

**Figure 3:**
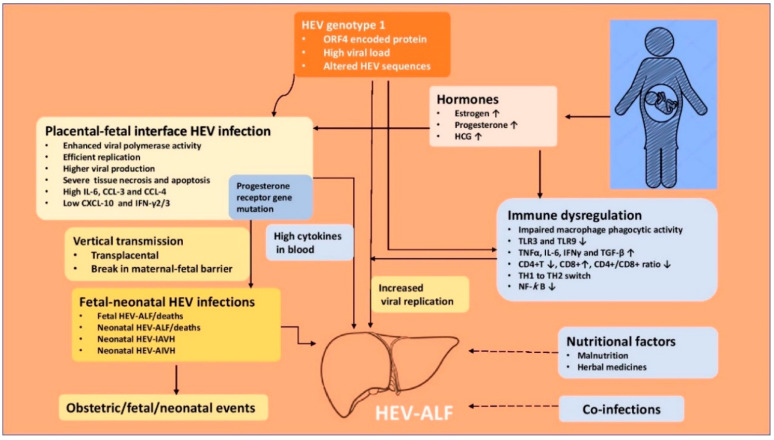
Pathogenesis of hepatitis E virus-related acute liver failure in pregnancy. IAVH = icteric acute viral hepatitis, AIAVH = Anicteric acute viral hepatitis, HEV-ALF = Hepatitis E virus related acute liver failure.

The pathogenesis of higher morbidity and mortality due to HEV infection in pregnancy is complex and remains to be fully understood. The lack of a proper animal model to study the pathogenesis of HEV-gt1 in pregnancy has been an issue. Rhesus monkey (Macaca mulata) is an established animal model for HEV infection [32,143], but HEV-gt1 infection to pregnant monkeys does not result in ALF [144,145] and thus is not useful to study the pathogenesis of the disease. Pregnant rhesus monkeys can be infected with HEV-gt4 with resultant high viral titers, longer duration of infection, obstetric events and vertically transmitted fetal infections [146]. A successful animal model for HEV-gt4 has been established in pregnant BABL/c mice [147]. Rabbits are new animal models for hepatitis E, but HEV-gt1 is not infectious to the animal [148]. Besides occurrence of ALF in pregnant women, extrahepatic manifestations including neurological, renal, hematological, and pancreatic are associated with HEV infections. The pathogenesis of HEV in the extrahepatic tissues is either due to direct cytopathic effect mediated by the virus replication or immunological mechanisms caused by an uncontrollable host response [149]. Several important facts have recently emerged to explain the complex relationship between HEV and pregnancy (Figure 3).

### 8.1. HEV Genome, Heterogeneity and Variants in Pregnancy

The data available from epidemic and sporadic hepatitis E indicate that pregnant women acquire HEV [19,126] and develop ALF more often than others [134,135,136] and this phenomenon is limited to infection with HEV-gt1 [59,65]. Other hepatitis viruses (HAV, HBV and HCV) and other HEV genotypes (HEV-gt2 and HEV-gt3) do not cause higher deaths in pregnant women [150]. Among the HEV family, HEV-gt1 alone has ORF4 which encodes a protein, pORF4 of 124 aa [38,39]. The encoded pORF4 by the HEV-gt1 genome interact with other viral and host proteins, enhance viral polymerase activity and promote viral replication [123]. HEV-gt1, and not HEV-gt3, causes necrosis and apoptosis in the maternal–fetal interface possibly caused by mitochondrial damage and activation of caspase family membranes [151]. This leads to alterations of the placental barrier architecture and promotes vertical transmission [38]. High levels of HEV RNA related to HEV-gt1 have been correlated with increased severity of disease in pregnant women [138,152]. HEV sequences in patients with HEV-ALF (five patients) were compared with those of HEV-AVH (five patients) and showed six unique amino acid substitutions in the ORF1 region of HEV [153], namely F179S, A317T, T735I, L1110F, V1120I, and F1439Y. Two of these (L1110F and V1120I) which occurred in the helicase domain pointing to its role in determining the outcome of HEV infections.

### 8.2. Immune Response in HEV Infected Pregnant Women

The immunological alterations in pregnancy are complex, showing immune tolerance to an allogenic fetus and host defense against pathogen. This is accomplished by the maternal–fetal interface (decidua) consisting of decidual stromal cells, decidual immune cells, and trophoblast cells [154]. The maternal–fetal interface contains natural killer (NK) cells, macrophages, dendritic, and T cells which interact with invading fetal extra-villous trophoblast for placentation, fetal growth, and pregnancy outcome [155]. Pregnancy suppresses cell-mediated immunity at the maternal–fetal interface to tolerate fetal antigens and maintains a normal humoral immune response against pathogens [156]. Pregnancy causes a shift from a Th1 to a Th2 cytokine response, allowing maternal–fetal tolerance for fetus development [157].

HEV-gt1 infection in pregnant women causes several alterations in innate and adaptive immune response, which helps virus replication and increases the severity of disease in the pregnant women [123]. Pregnant women with HEV-ALF have impaired macrophage phagocytic activity and downregulation of TLR3 and TLR9 expression, impeding MyD88-mediated IFN production [158]. This may lead to inadequate triggers for the innate immune response which in turn could lead to enhanced viral replication and severe liver injury. HEV-gt1 (but not HEV-gt3) in pregnant women was shown to evade early antiviral response and cause adverse consequences due to poor IFN response in placental cells [151]. Pregnant women with HEV-ALF have significantly higher levels of pro-inflammatory cytokines (TNF-alpha, IL-6, IFN-gamma, and TGF-beta) and this had a significant positive correlation with viral load, serum bilirubin, and prothrombin time. Increased severity of disease in pregnant women with HEV-ALF may be mediated through cytokines [159,160]. Pregnant women with HEV-ALF have lower CD4+ T cell counts, higher CD8+ T cell counts, and lowered CD4/CD8 ratio than women with HEV-negative ALF [161]. Pregnant women with HEV-ALF have excessive Th2 switching, which dysregulates balance between tolerance and immunity [162,163]. Pregnant women with HEV-ALF show higher DNA-binding activity of NF-κB and absence of p65 expression, leading to deregulated immunity and severe liver damage [164]. Recently the role of immune cells in the spread of HEV infection to various body organs has been studied. Immune cells are permissive to HEV and help the virus enter various body organs, as shown in vivo and in vitro studies [165,166]. It is possible that this may be one of the ways in which the virus reaches the maternal–fetal interface and causes vertical transmission.

### 8.3. Hormones and HEV in Pregnancy

Hormones in pregnancy may enhance HEV viral replication. Women experience a sudden and marked increase in estrogen and progesterone levels during pregnancy [167]. Estrogen helps the fetus to develop and mature. Progesterone suppresses the maternal immune response by stimulation of Th2 and reduction of Th1 cytokines, thereby preventing maternal rejection of the trophoblast [168]. Pregnant women with HEV infection have higher levels of estrogen, progesterone, and HCG than those without HEV infection. Higher hormone levels are apt to further dampen the immune response and help viral replication [155]. Estradiol has been shown to facilitate HEV replication in an in vitro experiment in A546 cells [169]. High estrogen in pregnancy causes placental dysfunction and leads to preterm delivery, low birth weight infants and fetal mortality [170]. Bose et al. [171] studied deregulation of the progesterone receptor signaling pathway in pregnant women with HEV-ALF, HEV infection, and healthy controls. Patients with HEV-ALF show progesterone receptor gene mutations and a high IL-12/IL-10 ratio and are associated with a poor maternal outcome.

### 8.4. Nutritional Status and HEV in Pregnancy

Malnutrition had been proposed to explain the reports of higher deaths with viral hepatitis in pregnancy in developing countries. However, the nutritional assessment of pregnant women in these reports had not been evaluated [13]. During the Gulmarg-Kashmir HEV epidemic 1978-79, caloric and protein intake as well as estimation of serum protein as an index of nutritional status in pregnant women were determined and compared with those in nonpregnant women (15–45 years) [19]. The caloric and protein intake and serum protein in pregnant women were within normal ranges (3242 + 551 cal/day, 60 + 20 g/day, and 3.3 + 0.4 g/dL respectively) and did not differ from those seen in nonpregnant women (300 + 450 cal/day, 50 + 15 g/day, and 3.2 + 0.6 g/dL; *p* > 0.50). The caloric and protein intake and serum proteins were well in acceptance with those recommended for Indian women [172]. Of significance was the observation that pregnant women who developed HEV-ALF had excellent nutritional status. Thus, malnutrition contributing to severe disease in pregnant women in developing countries was not collaborated by these data.

### 8.5. Fetal HEV Infections and Maternal Mortality, Obstetric Events and Neonatal Outcome

Morbidity and mortality among pregnant women and their neonates and obstetric events may be a reflection of the severity of the HEV infection in the mother [19,126,128]. However, there is growing evidence that vertically transmitted HEV infections causing fetal HEV infections may directly contribute to maternal mortality, obstetric events, and neonatal outcome [173]. Several investigators from India and the Middle East [140,174,175,176,177,178,179,180,181,182,183] have reported vertical transmission of HEV and resultant peri-natal morbidity and mortality (Table 3).

**Table 3:** Vertical transmission of HEV and maternal and obstetric events and neonatal outcome.

Author Year. [References]	HEV-PF	Maternal & Obstetric Events	HEV-Neonatal Status		Pattern of Neonatal HEV Disease	Outcome of HEV-Infected Neonates
			Babies	HEV-Infections		
Khuroo et al., 1995 [174]	10	ALF 6, Died 3, (DUD 2), FTD 7, PD 1.	8	6 (RNA 5, IgM 3, IgG-Seroconversion 1).	HEV-ALF 2, I-HEV 1, AI-HEV 3.	Died 2 (HEV-ALF, Liver biopsy 1 MHN), Recovered 4, RNA in 2 lasted 1 month.
Khuroo et al., 2009 [175]	26	ALF 15, Died 9 (DUD 5), FTD 15, PD 4, Ab 2.	19 (Died 1 due prematurity) + 2 aborted.	15 (RNA 10, IgM 12).	HEV-ALF 6, I-HEV 4, AI-HEV 5.	Died 6 (HEV-ALF, Liver biopsy 1 MHN with HEV RNA in liver), Recovered 9. RNA lasted for 4 weeks in 4, 8 weeks in 1, 32 weeks in 1. IgM lasted for 4 weeks in 3, for 8 weeks in 2.
Khuroo et al., 2006 [140]	36	ALF 16, DIC 9, Died 10, FTD 26, PD 7, Ab 3.	33 + 3 aborted.	25 (RNA 20, IgM 24).	HEV-ALF 14, I-HEV 9, AI-HEV 2.	Died 14 (HEV-ALF, Liver biopsy 14 MHN), Recovered 11.
Kumar et al., 2001 [177]	28	ALF 6, Died 3 (DUD 2), PD 2.	26	26 (RNA 26)	HEV-ALF 2, I-HEV 21, AI-HEV 3.	Died 2 (HEV-ALF), Recovered 24.
Singh et al., 2003 [176]	22	ALF 14, Died 14.	NK *, 6	3 (RNA 3).	I-HEV 1.	-
Kumar et al., 2004 [178]	28	ALF 9, Died 7, PD 18.	NK *, 18	6 (RNA 4, IgM 3).	-	-
Chibber et al., 2004 [179]	92	FTD 92 (Vaginal delivery 80, Caesarean in 12)	92	4 (RNA 4, IgM 4).	I-HEV 4 ***	-
El Sayed Zaki et al., 2013 [181]	9	9 **	9	9 (RNA 5, IgM 1, IgG 6).	RDS with icterus 5, I-HEV 3, Sepsis 1.	-
Sharma et al., 2017 [180]	144	ALF 41, Died 6 (DUD 6).	128	59 (RNA 15, IgM 59).	-	-
Bonney et al., 2012 [182]	3	ALF 2, Died 2 (DUD 1), PD 1, Ab 1.	1 + 1 aborted	1 (RNA 1, IgM 1).	I-HEV 1.	Recovered, RNA -ve 3 weeks., IgM -ve 4 weeks.
Pradhan et al., 2012 [183]	1	Fetal HEV-AVH 15 weeks.	1	1 (IgM cord blood, amniotic fluid & serum at birth).	Fetal ascites at 15 weeks pregnancy, resolved in follow up	Healthy baby delivered 38 weeks., LFT normal, IgM +ve.

PF = Pregnant females, ALF = acute liver failure, I-HEV = Icteric hepatitis E virus infection, AI-HEV= Anicteric hepatitis E virus infection, MHN = Massive hepatic necrosis, DIC = disseminated intravascular coagulation, FTD = full term delivery, DUD = mother died with baby undelivered, Ab = abortion, PD = premature delivery, RNA = HEV RNA +ve, IgM = IgM anti-HEV +ve, IgG = IgG anti-HEV +ve, RDS = respiratory distress syndrome. LFT = liver function tests, * = total babies born not known, ** = all deliveries had complicated clinical course, *** = baby developed icterus at 6 weeks of birth.

In a seminal piece of research, mother-to-child transmission of HEV in 10 pregnant women was studied [174]. Two mothers died with babies undelivered. Six of the eight live-born babies showed evidence of HEV infection at birth. HEV RNA was detected in cord and birth blood samples in five, IgM anti-HEV in three and IgG anti-HEV seroconversion in four babies. Two babies died within 24 h from hypothermia and hypoglycemia. A liver biopsy in one baby revealed massive hepatic necrosis. Of the remaining six, one baby had icteric hepatitis and the other three had anicteric hepatitis. In another study, vertical transmission of HEV in 15 of the 19 babies born to HEV-infected pregnant women was studied [175]. Six of these died within first week of life. The remaining nine babies survived and five showed HEV RNA for varying intervals lasting up to 32 weeks. All surviving babies recovered and none developed chronic liver disease. Recently the relationship of severity of disease in 36 pregnant women (HEV-AVH, 20 and HEV-ALF, 16) with the severity of HEV infection in fetuses and newborn babies was evaluated [140]. Babies born to HEV-ALF mothers were more often HEV infected, viremic, and born with severe disease than those with HEV-AVH. DIC in mothers with HEV-ALF occurred exclusively in pregnant women who delivered babies with HEV-ALF. All the six mothers who survived had delivered babies within 4 days (2.3 ± 1.0 days) of onset of encephalopathy. Based on these data, it was postulated that severe fetal disease is the likely cause of increased severity of HEV infection in the mother, akin to the mirror syndrome in pregnancy, and delivery of baby performed early in the course of the disease may reverse the severe maternal disease [184,185,186].

**Figure 4:**
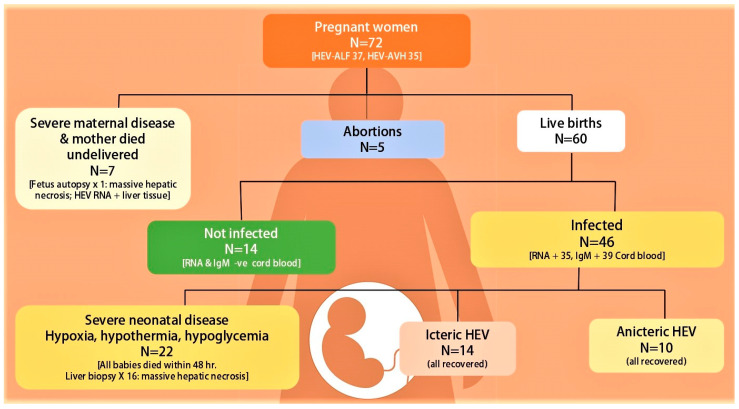
Fetal-neonatal hepatitis E virus infection recorded in fetuses and neonates from 72 pregnant women with hepatitis E virus infection seen at Sher-I-Kashmir Institute of Medical Sciences, Srinagar Kashmir, India from Dec 1993 onwards [140,174,175]. ALF = acute liver failure, RNA = HEV RNA, IgM = IgM anti-HEV.

Vertically transmitted fetal HEV infections and HEV infections in new-born babies have a wide spectrum of manifestations (Figure 4). Severe fetal disease results in intrauterine fetal death, and often the mother also had severe disease with DIC and died before delivery. Autopsies of such fetuses have shown massive hepatic necrosis. A significant proportion with severe disease present with hypothermia, hypoxia, and hypoglycemia at birth and die within 24 to 48 h of birth. Liver biopsies in such patients also show massive hepatic necrosis. Babies born with HEV infection may be asymptomatic with mild abnormality of liver tests (anicteric HEV) or present with jaundice with elevated live tests (Icteric HEV). The disease is self-limiting and serial follow-up reports clinical, serological and virological recovery in few weeks. A few babies show prolonged viremia lasting for up to 32 weeks, with eventual recovery.

### 8.6. Maternal–Fetal Interface HEV Infections

The role of HEV-gt1 is increasingly being recognized in causing infection and dysregulation at the maternal–fetal interface which leads to vertical transmission and increased systemic inflammation and consequent severe maternal disease [187]. Based on HEV transplacental transmission to the fetus, Bose et al. [188] studied placental HEV replication in 90 pregnant women (HEV-AVH 68 and HEV-ALF 22) and detected replicative HEV RNA and HEV RNA staining by ORF3, which correlated with fetal and maternal mortality in HEV-ALF patients. El-Mokhtar et al. [189] found that HEV-gt1 replicated more efficiently with a higher degree of inflammatory response in non-decidualized primary human endometrial stromal cells than in HEV-gt3. The authors believed that this infection mediates vertical transmission of HEV to the fetus. Recently, Gouilly et al. [151] infected ex vivo maternal–fetal interface with HEV-gt1 and HEV-gt3 and showed HEV-gt1 was more efficient than HEV-gt3 in viral replication and production and showed more severe tissue damage (necrosis and apoptosis) with upregulated IL-6, CCL-3, and CCL-4 and downregulated CXCL-10 and IFN-γ2/3. The authors concluded that HEV placental replication is genotype-specific and HEV-1 tropism at the maternal–fetal interface along with the extent of tissue damage, pro-inflammatory cytokines, and chemokines might be responsible for the severe maternal disease.

## 9. Management

HEV infection in pregnant women requires a team approach, determined by stage and severity of the disease in the mother, occurrence of obstetrical complications, and severity of vertically transmitted disease in the fetus/neonate.

HEV-AVH is usually a self-limiting disease and needs supportive medical treatment. At the onset, prodromal symptoms are limited to anorexia, fever, nausea, vomiting, and abdominal discomfort and generally subside within a week. A few patients may need a short hospital stay for intravenous fluids given for severe vomiting. Otherwise, patients are advised bed rest at home with bathroom privileges during prodrome and icteric disease. Later, physical activity is restricted and work can be resumed when disease recovery occurs. A portable high caloric diet with high carbohydrate and low fat is usually advised, but has no benefit in disease recovery. Cholestatic symptoms if intractable can be managed with antihistamines, cholestyramine, and/or ursodeoxycholic acid (UDCA). Corticosteroids should not be administered unless there is associated autoimmune hepatitis.

Patients are at high risk for ALF and the disease course can be unpredictable. So, a close watch on impending signs of liver failure (shrinking liver size, high or rising INR, rapid rise in serum bilirubin, and development of ascites or coagulopathy) must be kept. Patients with impending signs of ALF need intensive care management. Management policies to treat complications of ALF, namely encephalopathy, cerebral edema, hypoglycemia, coagulopathy, and possible DIC, GI bleed, sepsis, and renal failure, have been well standardized and should be immediately enforced. Liver transplantation team if available must be involved and considered if prognostic criteria employed are met [134,135,190,191]. Unfortunately, as of today, only isolated case reports of liver transplantation in pregnant women with HEV-ALF are published in the literature [192,193,194]

**Figure 5:**
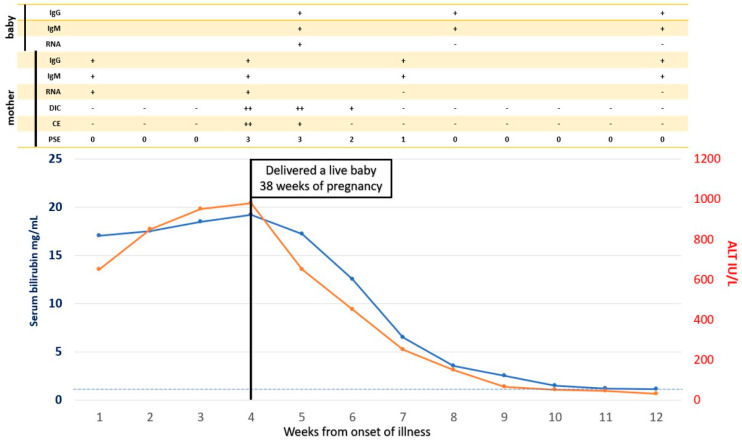
Spontaneous Vaginal Delivery in pregnancy with HEV-ALF and the outcome on maternal disease. A 30-year-old pregnant woman presented with features of icteric hepatitis E virus infection. On the 24th day of her illness, she had rapid deterioration with encephalopathy, cerebral edema, and laboratory features of DIC. She delivered a live baby vaginally with icteric hepatitis E four days after the onset of encephalopathy. Serial follow-up showed rapid clinical improvement in encephalopathy, cerebral edema, and DIC. PSE = portosystemic encephalopathy, CE = cerebral edema, DIC = disseminated intravascular coagulation, RNA = HEV RNA, IgM = IgM anti-HEV, IgG = IgG anti-HEV.

A close obstetric watch is needed in both HEV-AVH and HEV-ALF patients to evaluate the stage of pregnancy, fetal wellbeing, and growth. Complications such as abortions, preterm labor, premature rupture of membranes, stillbirth, intrauterine deaths, and increased risk of bleeding associated with coagulopathy can occur and need to be aggressively managed by standard obstetric guidelines [128]. Therapeutic termination of pregnancy and its beneficial effects on liver disease in the mother needs serious consideration [34,195], as events in the maternal–fetal interface and the fetus are involved and are incriminated in the severity of the maternal disease [151,184]. Therapeutic termination has proved effective in two other clinical syndromes, namely HELP syndrome and acute fatty liver in pregnancy, wherein fetal events drive the maternal disease [196,197]. Several investigators do not recommend termination of pregnancy in pregnant women with HEV-ALF [198,199]. This is based on the results of a retrospective study, in which 42 patients with HEV-ALF were studied [200]. Nine of the 22 women who delivered had died along with 14 of the women who continued their pregnancy. There was no significant difference in mortality in the two groups. However, in a subgroup analysis, the authors showed that patients with lesser degree (grade 1–3) of encephalopathy who delivered had lesser mortality (5/16) than those who continued their pregnancy (13/20, *p* < 0.046). Satia and Shilotri [201] from Mumbai, Maharashtra, India showed promising results of induction of labor in a pregnant woman with HEV-ALF with grade 2 hepatic encephalopathy and DIC. After induced vaginal delivery, the patient made an eventual recovery. The authors recommended therapeutic termination of pregnancy in HEV-ALF with lower grades of encephalopathy for a better maternal outcome. We showed that of the 16 pregnant women with HEV-ALF, 10 of whom also had DIC, all the six mothers (DIC in 3) who delivered babies within 4 days (mean ± 1SD 2.3 ± 1.0 days) from onset of encephalopathy and with lower grades of encephalopathy (mean ± 1SD 3.0 ± 0.89) survived. In contrast, all the 10 mothers, of whom six had DIC, who died had delivered babies after 4 days (mean ± 1SD 9.6 ± 3.0 days) and with higher grades of encephalopathy (mean ± 1SD 3.5 ± 0.53) (*p* = 0.02). The results of such an endeavor are striking in sick patients with severe metabolic problems and DIC (Figure 5). Thus, therapeutic or spontaneous termination of pregnancy with HEV-ALF early in the course of disease with lesser grades of encephalopathy was recommended [140]. Randomized trials on the therapeutic role of termination in HEV-ALF pregnant women need to be carried out. However, till the results of such trials are available, it is a safe practice to induce labor early in the course of disease with lower grades of encephalopathy for a better maternal outcome.

Neonatal care is essential as HEV is known to infect the fetus/neonate resulting in considerable fetal and perinatal morbidity and mortality [175].

Several drugs, namely ribavirin, pegylated IFN, and sofosbuvir, inhibit HEV replication, have an antiviral property, and are drugs of choice to manage chronic HEV-3 in solid organ transplant and patients with some hemopoietic malignancies [11]. Recently ribavirin has been successfully used to treat HEV acute-on-chronic-liver-failure and severe HEV infection in nonpregnant states [202]. Ribavirin is teratogenic and is contraindicated during pregnancy and there are no reports of ribavirin therapy in HEV-AVH and HEV-ALF.

## 10. Vaccination

The development of the HEV vaccine is seen as a breakthrough in the control of hepatitis E [203,204]. Vaccination against HEV in pregnant women and women of childbearing age in endemic regions may be the most important management strategy [158]. The Chinese vaccine, HEV 239 marketed as Hecolin [205], is protective against HEV-gt1. The vaccine has been shown to be safe in pregnant women [206] and protects against HEV infection and HEV-related adverse outcomes in pregnant rabbits [207]. Recently, a phase IV trial has been initiated to assess the effectiveness, safety, and immunogenicity of the HEV 239 vaccine in women of childbearing age in rural Bangladesh, where HEV infection is endemic [208]. Availability of the vaccine in developing countries is being watched with great interest [209].

## Abbreviations

AVH	Acute viral hepatitis
HEV	Hepatitis E virus
HAV	Hepatitis A virus
HCV	Hepatitis C virus
NANBH	non-A, non-B hepatitis
ET-NANBH	Enterically-transmitted non-A, non-B hepatitis
PT-NANBH	Parentally-transmitted non-A, non-B hepatitis
ENANBH	Epidemic non-A, non-B hepatitis
ALF	Acute liver failure
gt	genotype
ORF	Open Reading Frame
CFR	Case fatality rate
DIC	Disseminated intravascular coagulation
VLP	Virus like particles
